# Obesity: Next game changer of allergic airway diseases?

**DOI:** 10.1002/ctm2.70316

**Published:** 2025-05-07

**Authors:** Wenlong Li, Noah Marx, Qintai Yang, Deyu Fang, Yana Zhang

**Affiliations:** ^1^ Department of Otolaryngology‐Head and Neck Surgery The Third Affiliated Hospital of Sun Yat‐Sen University Guangzhou China; ^2^ Department of Allergy The Third Affiliated Hospital of Sun Yat‐Sen University Guangzhou China; ^3^ Department of Otolaryngology‐Head and Neck Surgery Key Laboratory of Airway Inflammatory Disease Research and Innovative Technology Translation Guangzhou China; ^4^ Naso‐Orbital‐Maxilla and Skull Base Center The Third Affiliated Hospital of Sun Yat‐Sen University Guangzhou China; ^5^ Department of Pathology Northwestern University Feinberg School of Medicine Chicago Illinois USA

**Keywords:** allergic airway diseases, biologics, endotype, obesity, therapeutic resistance

## Abstract

Obesity and allergic diseases are global health concerns, both of which are seeing an increase in prevalence in recent years. Obesity has been recognised as an important comorbidity in subpopulations with allergic airway diseases, which represents a unique phenotype and endotype. Obesity‐related allergic airway diseases are associated with exacerbated clinical symptom burden, altered immune response, increased disease severity and compromised predictive capability of conventional biomarkers for evaluating endotype and prognosis. Moreover, treatment of obesity‐related allergic airway diseases is challenging because this unique endotype and phenotype is associated with poor response to standard therapeutic strategies. Therapeutic regimen that involves weight loss by non‐surgical and surgical interventions, gut microbiome‐targeted treatment, glucagon‐like peptide‐1 receptor agonist and other agents should be considered in this population. In this review, we outline the current knowledge of the impact of obesity on prevalence, endotypes, clinical symptom and management of allergic airway diseases. Increased understanding of the implications of obesity may contribute to better treatment options for the obesity‐related refractory airway inflammation, particularly in precision medicine.

**Key points:**

- Obesity can increase the prevalence of allergic airway diseases such as asthma, AR, and CRSwNP.
- Obesity alters the immune endotype and exacerbates clinical symptoms of respiratory allergic diseases.
- Obesity‐related allergic airway diseases exhibit therapeutic resistance to standard treatment.
- Obesity‐related allergic airway diseases constitute a distinct category of endotypes and phenotypes, requiring further in‐depth research and novel therapeutic approaches.

## Graphical abstract

Obesity affects various aspects of allergic airway diseases, including prevalence, endotypes, clinical features and therapeutic efficacy. Therefore, obesity‐related allergic airway diseases represent a unique class of endotypes and phenotypes, which need much more in‐depth research for tailored treatment.

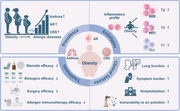

## 1 INTRODUCTION

The prevalence of obesity and allergy has increased worldwide over the last decades, leading to the hypothesis that these two non‐communicable diseases might be interconnected.^1^, ^2^, ^3^ The comorbidity of obesity in subpopulations with allergic diseases, such as asthma, chronic rhinosinusitis (CRS) and allergic rhinitis (AR), has recently been identified as a unique endotype and phenotype.^4^, ^5^, ^6^ Emerging evidence suggests that obesity can influence the pathophysiology of allergic upper and lower airway diseases through various mechanisms, including systemic inflammation, altered immune responses and changes in respiratory mechanics. Furthermore, clinical and experimental data demonstrate that the obesity‐associated chronic low‐level inflammation contributes to exacerbated airway inflammation, difficult‐to‐treat symptoms and therapeutic resistance in allergic individuals.

While many factors can contribute to allergic airway inflammation, the role of metabolic disorders, particularly obesity, needs more research. Furthering our understanding of the complex underlying mechanisms and impact of obesity will help provide deeper insights into the pathophysiology of respiratory allergic diseases and may support ambitions of precision medicine in the management of those patients, as well as broader goals to elevate patient care. Here, we review the emerging evidence for obesity's impact on respiratory allergic diseases, with a spotlight on the therapeutic resistance. In addition, we show how an improved understanding of obesity can be used to develop therapies to treat atopic airway diseases, and we discuss the potential of new and existing therapeutics in improving clinical burden in obese populations with allergic airway diseases.

## 2 OBESITY INCREASES THE PREVALENCE AND INCIDENCE OF ALLERGIC AIRWAY DISEASES

Factors such as gene polymorphisms,^7^ environmental exposures,^8^ diet^9^ and microbiome^10^ have been reported to contribute to obesity‐related airway allergic diseases. Although how obesity changes the architecture of allergic immune responses is not well defined, there is mounting evidence that obesity may increase the prevalence and incidence of allergic airway diseases including asthma, AR and CRS, both in adults and children.

Generally, obesity/overweight is considered as a risk factor for adult asthma. A meta‐analysis including 16 cohort studies has concluded that elevated body mass index (BMI), increased waist circumference and weight gain are strongly associated with increased risk of developing asthma in adults. Also, the same study has revealed a 32% increase in the relative risk of developing asthma per 5 kg/m^2^ elevation in BMI through a linear dose–response analysis.^11^ Similarly, the positive relationship between obesity and increased prevalence of allergic upper airway diseases such as AR and CRS has also been observed in a cross‐sectional analysis from the United States.^12^ Further studies from Korea, China and the United States have confirmed that obesity/overweight tightly correlates with increased prevalence of AR (odds ratio [OR] = 3.1, 95% confidence Interval [CI]: 1.1–8.7), CRS (OR = 1.33, 95% CI: 1.17–1.51) and CRS with nasal polyps (CRSwNP; OR, 1.438, 95% CI: 1.170–1.768).^13^, ^14^, ^15^ However, data noted in the literature regarding the association between obesity and AR in adults are inconsistent. Some studies suggest that there is no association between overweight/obesity and AR in adults.^16^, ^17^ Conversely, it seems that obesity is linked to increased odds of non‐AR in adult male subjects (adjusted OR = 1.43, 95% CI: 1.06–1.93).^18^ These conflicting data indicate that AR has a complex pathogenesis, and metabolic dysregulation may be just one of the causes.

Obesity/overweight may also exert an influence on the increased incidence of asthma and AR in children. A meta‐analysis of 15 epidemiological studies has revealed that childhood obesity significantly elevated the odds of asthma by about 50% compared with those without obesity (OR 1.5, 95% CI: 1.3–1.7).^19^ A similar association has also been found in overweight children with asthma.^20^ An early study (2006–2008) did not identify a significant association between obesity and AR in children.^21^ Recently, a meta‐analysis including 30 studies published in 2020 has found a significant correlation between AR and overweight/obesity in children (OR = 1.09, 95% CI: 1.06–1.12).^16^ Unlike in adults, no association has been observed between obesity and CRS in children,^14^, ^21^ implying heterogeneity in pathogenesis of allergic respiratory diseases between adults and children.

Interestingly, maternal obesity/overweight may increase the incidence of allergy in children. Growing evidence for the effect of maternal obesity/overweight on childhood asthma has emerged.^22^, ^23^ Furthermore, a cross‐sectional study evaluating 8877 mother–child pairs has shown that higher gestational weight gain in mothers correlates with the increased risk of childhood allergic diseases like asthma and AR,^24^ which indicated that susceptibility to allergies in a child may originate as early as the foetal period.^25^ Further research on the long‐term effects of the intrauterine environment on children's health may need to take maternal weight management during pregnancy into consideration since gestational weight gain could be a controllable and modifiable risk factor.^24^

## 3 OBESITY ALTERS IMMUNE ENDOTYPES

The most commonly accepted and well‐defined endotype of allergic diseases is one encompassed by eosinophilic inflammation resulting from type 2 (T2) cells including CD4+ T helper 2 (Th2) cells, group 2 innate lymphoid cells (ILC2s), type 2 cytotoxic T (Tc2) cells and mast cells as well as T2 inflammatory mediators such as interleukin (IL) ‐4, IL‐5, IL‐13 and immunoglobulin E (IgE).^26^ Mounting evidence has demonstrated that obesity amplifies type 2 inflammation. Compared with asthmatic patients without obesity, those subjects with obesity exhibit elevated sputum IL‐5 and submucosal eosinophils.^27^ Meanwhile, increased capabilities of eosinophil adhesion and chemotaxis were found in asthmatic children and adolescent patients with obesity.^28^ Consistent with this, enhanced eosinophil uptake shown by in vivo imaging was reported in obese asthmatic patients compared with non‐obese asthmatics.^29^ In contract to obesity, overweight seems not result in increased eosinophilic inflammation in asthma patients. Overweight asthmatic patients demonstrate comparable eosinophil counts (peripheral blood, bronchial submucosa and sputum) and sputum IL‐5 levels to those with normal BMI.^6^, ^27^ Similarly, a study in children and adolescents has reported that overweight does not alter blood eosinophil counts or levels of total IgE, IL‐4 or fractional exhaled nitric oxide (FeNO).^30^ In addition to eosinophils, increased proliferation and survival of Th2 cells and ILC2s induced by leptin was observed in mouse models of allergic airway disease and allergic patients with obesity.^31^, ^32^ Moreover, hyperactivated eosinophils and Charcot‐Leyden crystals were elevated in overweight/obese CRSwNP subjects compared with those with normal BMI, indicating exacerbated eosinophilic inflammation in CRSwNP patients with higher BMI.^4^

Recently, emerging evidence has suggested an important role for obesity in converting typical T2‐biased inflammation to type 3 (T3) predominant response in allergic subjects.^5^ T3 immunity is mediated by group 3 innate lymphoid cells (ILC3s), CD4+ T helper 17 (Th17) cells and type 17 cytotoxic T (Tc17) cells, which are activated by IL‐1β and IL‐23 and subsequently produce IL‐17 families of cytokines, resulting in neutrophil infiltration.^33^ Reduced eosinophil infiltration and T2 biomarkers were observed in obese patients with refractory asthma or CRS,^6^, ^34^ suggesting the existence of a T2‐low inflammation phenotype in obese patients. Conversely, elevated neutrophil counts and sputum and plasma IL‐17 concentration have been observed in obese asthma subjects, which was associated with poor asthma control and higher doses use of inhaled corticosteroids.^35^, ^36^, ^37^ Similarly, high plasma and sputum IL‐6 levels were associated with increased BMI and neutrophil count in both child and adult asthmatics, which may drive worse pulmonary function and more severe clinical symptoms.^38^, ^39^ Meanwhile, neutrophil^high^eosinophil^high^ inflammatory profile associated with increased recurrence was identified in overweight/obese CRSwNP patients.^4^ Interestingly, inflammatory endotype shifting from T2 to T3 seems more common in obese patients with atopy compared with those without atopy. A cross‐sectional study from Western China demonstrated obesity increased sputum macrophage counts, with a decreased tendency in sputum eosinophils and serum IL‐5 levels in the atopic asthmatics, which was not observed in non‐atopic patients.^40^ Consistent with asthma, data from mouse models of atopic dermatitis demonstrated that obesity converted the classical TH_2_‐predominant disease to a more severe disease with prominent TH_17_ inflammation.^5^

**FIGURE 1:**
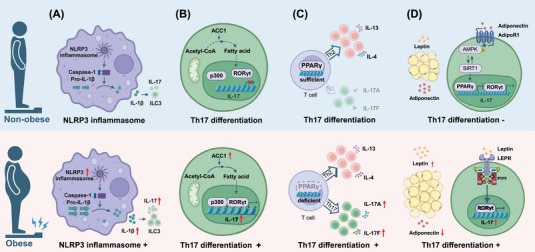
Molecular mechanisms driving increased type 3 inflammation in obese patients with allergic airway diseases. (A) Obesity‐induced NLRP3 inflammasome activation facilitates infiltration of ILC3s and subsequent IL17 secretion. (B) Obesity augments Th17 cell differentiation by inducing of ACC1 and fatty acid synthesis. (C) Obesity alters the inflammatory endotype from T2 to T3 by inducing a deficiency of transcription factor PPARγ in T cells. (D) Leptin can promote RORγt transcription via the JAK2/STAT3 pathway, whereas adiponectin can suppress RORγt through the AMPK/SIRT1/PPARγ pathway. Elevated leptin and reduced adiponectin levels in obese patients exacerbate T3 inflammation. ACC1, acetyl‐CoA carboxylase 1; adipoR1, adiponectin receptor 1; AMPK, AMP‐activated protein kinase; IL, interleukin; ILC3, Innate lymphoid cell type 3; JAK2, Janus kinase 2; LEPR, leptin receptor; NLRP3, nucleotide oligomerisation domain‐like receptor protein 3; PPARγ, peroxisome proliferator‐activated receptor γ; RORγt, retinoid‐related orphan nuclear receptor γt; SIRT1, sirtuin 1; STAT3, signal transducer and activator of transcription 3. Figure was created with BioRender.

It has been hypothesised that obesity could promote type 3 inflammation in allergic airway diseases through various mechanisms (Figure 1). First, the domain‐like receptor protein 3 (NLRP3) inflammasome plays an important role in linking obesity to T3 inflammation. Clinical data and animal experiments found that obese asthmatic patients and murine model had significantly increased NLRP3 inflammasome activation and levels of IL‐1β.^9^, ^41^ Activation of the NLRP3 inflammasome in the lungs not only facilitates the infiltration and activation of neutrophils, but also promotes the expansion and activation of ILC3s and IL17A secretion, which contribute to exacerbated asthma symptoms and increased steroid resistance.^4^, ^9^, ^41^ In contrast, deletion of NLRP3 reverses the symptoms in mouse models of obese asthma,^42^ suggesting targeting NLRP3 inflammasome has important potential in obesity‐associated airway allergic diseases. Second, transcription factor retinoid‐related orphan receptor gamma t (RORγt) is essential for the differentiation of Th17 cells, which could be modified by fatty acid synthesis.^43^ A study in high‐fat‐fed mice demonstrated that obesity could augment Th17 differentiation through modulating RORγt function by inducing of the acetyl‐CoA carboxylase 1 (ACC1, the gene product of *ACACA*) and fatty acid synthesis in vivo and in vitro.^44^ A strong correlation between TH17 cells and *ACACA* expression levels was also observed in obese human subjects in their study. Third, compromised expression of transcription factor peroxisome proliferator‐activated receptor‐γ (PPARγ) may favour Th17 differentiation. Recently, authors from Salk Institute found that obese animals with allergic dermatitis had substantial Th17‐biased inflammation as well as the canonical Th2‐skewed response seen in lean counterparts, suggesting obesity could alter the inflammatory endotype from T2 to T3 and worsen obesity‐related conditions. The possible reasons have been hypothesised that PPARγ protects the dominance of Th2 response, which may be damaged and convert to Th17 response during obesity. Therefore, the maintenance of Th2 responses by PPARγ seems to prevent the amplification of a more pathological Th17‐biased inflammation, suggesting PPARγ agonists could be used for targeting obesity‐related allergic diseases.^5^ Fourth, adipokines such as leptin and adiponectin derived from adipose tissue play a significant role in the regulation of obesity‐associated T3 inflammation. Elevated leptin levels and Th17 cells were observed in obese asthmatic patients and obese children with AR.^45^, ^46^, ^47^ Studies in mice models demonstrated that leptin could drive Th17 cells differentiation by promoting glycolytic metabolism and enhancing RORγt expression in T cells.^48^, ^49^ In addition, leptin has been reported to attract neutrophil infiltration in vitro by facilitating IL‐8 secretion in lung fibroblasts and activating mitochondrial reactive oxygen species (mtROS)–NLRP3 inflammasome in airway epithelial cells, respectively.^50^, ^51^ In contrast, adiponectin and its receptor agonist have been shown to alleviate neutrophilic inflammation likely by modulating AMP‐activated protein kinase (AMPK) signalling pathway in a murine model of experimental allergic airway disease.^52^, ^53^ This may explain why obese asthmatics with adiponectin deficiency exhibited enhanced neutrophilic inflammation in the lungs.^54^

**FIGURE 2:**
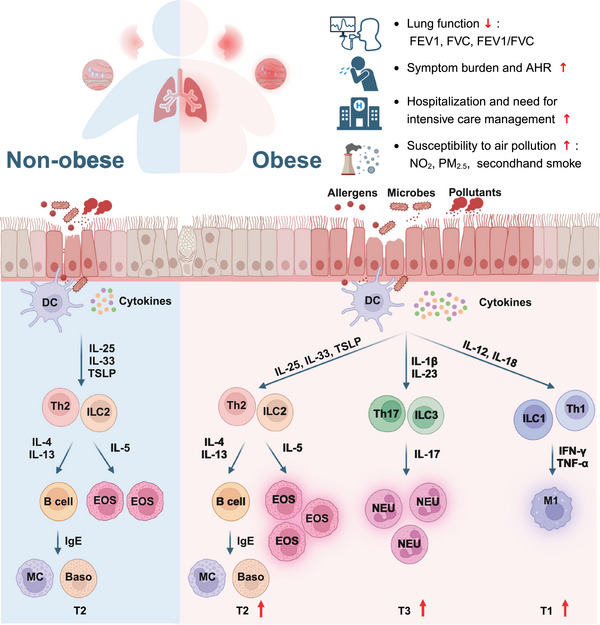
Overview of the inflammatory profiles between obese and non‐obese patients with allergic airway diseases. Typically, allergic airway diseases are characterised by a predominant T2 immune response. Obesity could enhance the T2 inflammation and alter the inflammatory endotype from T2 to T3 or T1. Accordingly, obese patients may suffer from more severe clinical burden in many aspects. Baso, basophil; DC, dendritic cell; EOS, eosinophil; FEV_1_, forced expiratory volume in one second; FVC, forced vital capacity ratio; IFN‐γ, interferon gamma; IgE, immunoglobulin E; IL, interleukin; ILC1, group 1 innate lymphoid cell; ILC2, group 2 innate lymphoid cell; ILC3, group 3 innate lymphoid cell; M1, classically activated macrophage; MC, mast cell; NEU, neutrophil; NO_2_, nitrogen dioxide; PM_2.5_, fine particles less than 2.5 µm in diameter; T1, type 1; T2, type 2; T3, type 3; TNF‐α, tumour necrosis factor alpha; TSLP, thymic stromal lymphopoietin. Figure was created with BioRender.

In addition, a meta‐analysis from South Africa suggested that the inflammation in obesity‐related asthma in children was characterised by Th1‐mediated immune response rather than Th2‐mediated immunity.^55^ The discrepancy in endotype implies heterogeneity in inflammatory profile between children and adults. Since impaired predictive capability of conventional T2 biomarkers has been reported in obese asthmatics and CRSwNP patients, there is an urgent need for novel biomarkers predicting immune endotype and disease prognosis.^4^, ^6^ The inflammatory cell profiles between obese and non‐obese patients with allergic airway diseases has been summarised in Figure 2. The implication and significance of identifying the altered endotype in obese allergic individuals is to facilitate tailored precision treatment and provide appropriate predictive biomarkers for those patients.

## 4 OBESITY EXACERBATES CLINICAL BURDEN

Patients with obesity not only have an increased risk of developing allergic respiratory diseases, but also manifest more severe and difficult‐to‐control clinical symptoms. Compared with non‐obese patients with allergic respiratory disease, obese individuals exhibit higher symptom scores and medication scores, as well as elevated frequency and length of respiratory‐related hospitalisations.^46^, ^56^, ^57^, ^58^

Recent studies have shown that obesity is detrimental to lung function. Decreased forced expiratory volume in one second (FEV_1_), forced vital capacity ratio (FVC), total lung capacity and residual volume were observed in adult asthmatics with higher BMI compared with those with normal weight.^6^, ^59^ This is likely attributed to fat deposition in the chest and abdomen, leading to reduced thoracic expansion and diaphragmatic mobility, and subsequently decreased respiratory compliance and increased respiratory work.^42^ Compared with adults, asthmatic children with obesity exhibited more pronounced FEV_1_/FVC deficit with unchanged FEV_1_ or FVC, indicating that obesity has heterogeneous effects on lung function between child and adult asthmatics.^57^, ^59^ One potential explanation for this is the mismatch between a faster growth in airway length and the slower expansion in airway caliber.^60^ In addition to impaired baseline lung function, lower prevalence (35%) of reversible airflow obstruction by either ipratropium or albuterol was found in obese asthmatics.^6^ Interestingly, lower FeNO levels have been observed in obese versus non‐obese asthmatics (both adults and children),^6^, ^36^, ^61^, ^62^ suggesting that the exacerbated clinical symptoms may be driven by increased T3 inflammation. In contrast, some studies involving obese adults and paediatrics with asthma did not find any correlation between FeNO and obesity.^30^, ^63^ These contrasting results may support the hypothesis that there are different subphenotypes of obese asthma. Compromised lung function was also reported in obese AR patients. Lower forced mild‐expiratory flow (FEF_25–75_), FEV_1_ and FEV_1_/FVC were observed in AR patients with high BMI compared with those with normal weight, suggesting that AR patients with obesity are more susceptible to bronchial impairment that may evolve to asthma.^64^ In addition, compared with non‐obese children with AR, obese AR children present higher symptom scores and medication scores.^46^, ^56^ Whether obese childhood AR individuals are more susceptible to asthma needs further study. Beyond impaired lung function, obesity has been reported to enhance airway hyperresponsiveness (AHR) in asthmatic patients, which may be associated with inflammatory status induced by obesity.^6^, ^65^, ^66^, ^67^ Impaired lung function and increased AHR may contribute to severe persistent symptoms and less remission.^68^ As a severe and difficult‐to‐treat phenotype, obese asthmatics exhibited an elevated number of annual symptom exacerbations, increased frequency and length of respiratory‐related hospitalisations and increased risk of mechanical ventilation use as well as the need for intensive care unit (ICU) level care.^57^, ^58^, ^69^, ^70^ Overweight asthmatic patients (both adults and children) also experience worse clinical outcomes than normal‐weight counterparts, with higher risk of asthma‐related hospitalisations, emergency department visits and extended high‐dependency unit stays.^57^, ^71^ Meanwhile, a retrospective cohort study of asthmatic pregnant women has found that obesity could increase the risk of adverse perinatal outcomes like late preterm birth, perinatal death and ICU admissions, highlighting the importance of accounting for BMI control during pre‐conception in obese female asthmatics.^72^ Of note, a previous National Health and Nutrition Examination Survey study revealed stronger correlations between obesity and clinical outcomes in non‐atopic children versus atopic counterparts, with non‐atopic children demonstrating higher odds of current asthma, asthma attack and wheezing episodes.^73^ However, later study demonstrated that atopy significantly mediates the effect of adiposity on asthma outcomes in children asthmatics.^74^ This discrepancy may be due to differences in disease severity and asthma endotypes.

CRS subjects with obesity exhibited increased incidence of nasal obstruction, purulent discharge and olfactory dysfunction.^4^, ^15^ Obesity was also identified as one of the significant risk factors of habitual snoring in CRS, perhaps because fat deposits around the upper airway could worsen airway collapse.^75^ CRSwNP patients with obesity also showed increased postoperative recurrence rate due to augmented eosinophilic–neutrophilic inflammation.^4^, ^76^

Interestingly, it seems that obesity may amplify the effect of air pollution on allergic diseases. Increased BMI made asthmatic children and AR children more vulnerable to air pollutants such as nitrogen dioxide (NO_2_), fine particles less than 2.5 µm in diameter (PM_2.5_), fine particles less than 10 µm in diameter (PM_10_) and second‐hand smoke exposure, presenting with the increased odds of having an asthma symptom and worse nasal discomfort.^77^, ^78^, ^79^, ^80^ Moreover, high BMI was associated with the combined symptoms of anxiety and depression in severe asthma,^81^ and these psychological disorders also correlated with the levels of asthma control,^82^ indicating the necessity of screening and treating psychological disorders in these patients.

## 5 OBESITY CONTRIBUTES TO THERAPEUTIC RESISTANCE

**FIGURE 3:**
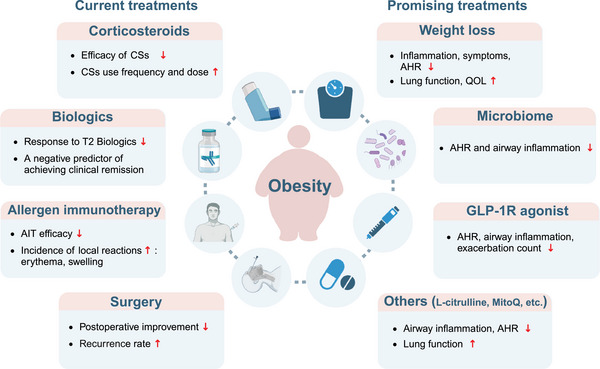
Obesity‐associated treatment insensitivity and promising therapies for patients with obesity‐related allergic airway diseases. Obesity can reduce the efficacy of current treatments including corticosteroids, biologics, allergen immunotherapy and surgery in patients with allergic airway diseases. Thus, it is crucial to find novel therapeutic strategies for these patients. Weight loss, microbiome‐targeted therapy, GLP‐1R agonists and other potential therapies may be beneficial for obese patients with allergic airway diseases. AGs, 1‐*O*‐alkyl‐glycerols; AHR, airway hyperresponsiveness; AIT, allergen immunotherapy; CSs, corticosteroids; GLP‐1R, glucagon‐like peptide‐1 receptor; QOL, health‐related quality of life; T2, type 2. Figure was created with BioRender.

Obesity can change typical T2 inflammation to T3 inflammation in allergic individuals, which may convert an effective therapy to an anti‐therapy. Growing clinical evidence has shed light on the important role of obesity for treatment resistance in allergic patients (Table 1 and Figure 3).

**TABLE 1:** Efficacy of various therapies on overweight/obese patients with allergic airway inflammation.

Management	Diseases	Manifestation	Allergen tests
**Corticosteroids**			
Inhaled corticosteroids (ICS)	Asthma	Less improvement in lung function in overweight/obese subjects compared with those with normal weight^83^	Skin test
		Lower improvements in total daily asthma symptom scores and FeNO in overweight group compared with those with normal weight^84^	NA
		Increased ICS use and dose in obese asthmatics compared with healthy‐weight subjects^85^	NA
		Decreased response to inhaled budesonide in obese children based on FEV_1_/FVC and urgent care visits/hospitalisations^86^	NA
		A linear decrease in asthma control with increasing BMI in atopic asthma, but not in non‐atopic asthma^87^	skin tests, serum IgE, blood eosinophilia, history of atopy
		The use of ICS did not differ significantly between overweight and normal‐weight asthmatics^58^	SPT
		Preschool children responded well to ICS when measured by daily asthma symptoms and exacerbations^88^	SPT or sIgE
Internasal corticosteroids (INCS)	AR	No significant differences in the improvement of questionnaire scores regarding nasal symptoms were observed between obese and normal weight subjects, while IL‐10 levels were significantly lower in the obese group^89^	SPT
Oral corticosteroids (OCS)	Asthma	The use of OCS in obese asthmatic subjects is higher than those with normal weight^85^	NA
		No significant increase was found in the use of OCS between overweight and normal‐weight asthmatics^58^	SPT
		A significant reduction in prednisone absorption and an increase in clearance were found when BMI increased in asthmatic subjects^90^	SPT
	CRS	High BMI was a risk factor for oral corticosteroids insensitivity in CRSwNP (OR 1.584, 95% CI: 1.002–2.505), particularly in eosCRSwNP subjects^91^	SPT or sIgE
**Other conventional medications**			
Short‐acting β2‐agonists (SABA)	Asthma	Obese asthmatic children and adolescents are more likely to be non‐responsive to SABA compared with their non‐obese counterparts (OR 1.24, 95% CI: 1.03–1.49)^92^	NA
Long‐acting β2‐agonists (LABA)	Asthma	Obese patients are less likely to achieve control compared with those with normal BMI^87^	skin tests, serum IgE, blood eosinophilia, history of atopy
add‐on therapies	Asthma	More add‐on therapies (long‐acting β2‐agonist, leukotriene receptor antagonist, theophylline or antimuscarinic agents) were used in obese or overweight asthmatics^58^	SPT
**Biologics**			
Omalizumab	Asthma	Obese patients were less likely to be classified as responders than non‐obese patients (41 vs. 66%, *p* = .017). However, in those patients who did respond to omalizumab, the average effect on ACQ‐5 was similar in obese and non‐obese patients^93^	sIgE or allergen history
		Obesity could lead to a reduced effectiveness to omalizumab treatment based on higher number of exacerbations, a reduced ACT increase and a lower asthma control^94^	SPT
		Significantly improved lung function and asthma control, reduced rescue medication and asthma exacerbations were found in obese subjects^95^	NA
		Improved lung function and asthma symptom score, reduced asthma exacerbations and corticosteroids dose were found across all BMI categories^96^	SPT
Benralizumab	Asthma	Asthmatic patients with higher BMI were less likely to reduce exacerbation rates^97^	Phadiatop test
		Higher BMI is a poor response to benralizumab in asthmatic patients^98^	SPT, tIgE, blood eosinophil counts, and FeNO levels
		Improvements in pre‐bronchodilator FEV_1_ and reduced exacerbations were observed across all BMI groups^99^	NA
	CRS	Less amelioration of nasal polyp score and nasal blockage score was observed in CRS subjects with high BMI^100^	Phadiatop test
Mepolizumab	Asthma	The average BMI in the non‐remission group is higher than that in the clinical remission group^101^	NA
		BMI is a negative predictor of achieving clinical remission (OR 0.91, 95% CI: 0.85–0.96)^102^	NA
		BMI is not a predictor of treatment response (OR 1.03, 95% CI: 0.95–1.12)^103^	NA
		Decreased annual rate of clinical exacerbations and reduced blood eosinophil counts were observed across all BMI groups^104^	NA
		Reduced exacerbations were observed in both obese and non‐obese patients, while the obese group showed less improvement in lung function compared with the non‐obese group^105^	NA
Dupilumab	Asthma	The annualised rate of severe exacerbations was significantly reduced across all BMI groups^106^	NA
		The proportion of obese subjects in the non‐remission group is higher than that in the clinical remission group (85.7 vs. 14.3%), a negative predictive factor for the treatment^107^	SPT
	CRS	Nasal congestion, nasal polyp score, University of Pennsylvania Smell Identification Test, loss of smell, Lund‐Mackay CT scan score and SNOT‐22 were significantly improved regardless of BMI^108^	NA
**Allergen immunotherapy**			
Sublingual immunotherapy (SLIT)	AR	The proportion of good responders in the BMI ≥ 25 group was lower compared with the BMI < 25 group in AR (Japanese cedar pollen extract)^109^	sIgE, family history of allergic diseases
		Improvements in symptom and medication scores were not correlated with BMI in children with AR (Dermatophagoides farinae drops)^110^	SPT or sIgE
Subcutaneous immunotherapy (SCIT)	Asthma	Lower improvement in FEV_1_/FVC was observed in obese asthmatic patients compared with those with normal weight^111^	NA
	AR	BMI (OR 1.506; 95% CI: 1.091–2.079) was a risk factor for local reactions in children with AR (house dust mite)^112^	SPT or sIgE
		No significant difference in BMI was observed between the effective and ineffective clinical response groups in AR (house dust mite)^113^	SPT
**Surgery**			
Endoscopic sinus surgery (ESS)	CRS	The percentage of relative improvement in QOL, as evaluated by RSDI and SNOT‐22 scores, may be decreased in obese patients compare with non‐obese patients^114^	History or modified radioallergosorbent testing
		Obesity was associated with decreased odds of surgical complications such as perioperative bleeding^115^	NA
		Higher postoperative sinonasal outcome test scores were found in obese CRSwNP subjects^34^	Other allergic disease: AR
		High BMI could increase the risk of recurrence in CRS subjects post‐surgery and BMI is one of the risk factors of CRS recurrence^76^	Other allergic disease: AR
		Increased recurrence of non‐eos CRSwNP was observed in the high BMI group compared with the underweight and normal weight group^91^	SPT or sIgE
		Increased BMI could enhance recurrent rate of non‐eos CRSwNP^4^	sIgE
Bronchial thermoplasty (BT)	Asthma	There is no relationship between baseline BMI (from 19 to 51 kg/m^2^) and improvement in ACQ score and FEV_1_ after BT^116^	NA
		A significant decrease in severe exacerbations, emergency department visits and hospitalisations was found in different BMI subgroups after BT^117^	NA
		The asthma quality of life questionnaire scores were more improved in patients with higher BMI than those with normal weight after BT^118^	sIgE

Abbreviations: ACQ, asthma control questionnaire; ACT, asthma control test; AR, allergic rhinitis; CI, confidence interval; CRS, chronic rhinosinusitis; CRSwNP, chronic rhinosinusitis with nasal polyps; eosCRSwNP, eosinophilic chronic rhinosinusitis with nasal polyps; FeNO, fractional exhaled nitric oxide; FEV_1_, forced expiratory volume in the first second; FVC, forced vital capacity; NA, not available; non‐eos CRSwNP, non‐eosinophilic chronic rhinosinusitis with nasal polyps; OR, odds ratio; QOL, quality‐of‐life; RSDI, rhinosinusitis disability index; sIgE, serum allergen‐specific IgE; SNOT‐22, the 22‐item sino‐nasal outcome test; SPT, skin prick test; tIgE, total immunoglobulin E. The atopy status was evaluated based on serum IgE level, skin prick test, blood eosinophilia, FeNO and documented history of atopy.

### 5.1 Corticosteroids

Corticosteroids (CSs), as the mainstay of allergic diseases therapy, exert their therapeutic effects by suppressing of inflammatory signalling. Increasing clinical evidence has suggested that obesity was associated with blunted response to CSs in allergic patients.^4^, ^83^, ^91^ Compared with asthmatic patients with normal weight, asthmatic adults and children with high BMI displayed a decreased response to inhale corticosteroids (ICS) as indicated by smaller improvements in FEV_1_, FEV_1_/FVC, unimproved rate of emergency department visits and less amelioration of asthma symptom scores.^83^, ^84^, ^86^ Intriguingly, a study has found that the response to ICS exhibits a linear decline with increasing BMI in atopic asthma, whereas the tendency is absent in non‐atopic asthma.^87^ This may be due to the fact that non‐atopic asthma patients are less responsive to ICS. In addition, increased use frequency and dose of ICS and even oral CSs were reported in obese subjects aiming to control asthma.^85^ Further, diminished responses to the combination of ICS and long‐acting β2‐agonists (LABA) such as salmeterol in adults,^87^ and to short‐acting β2‐agonists like salbutamol among children^92^ were reported, suggesting reduced efficacy of ICS in obese asthmatics could not be rescued by administration of additional β‐agonists. Overweight asthmatics also showed higher utilisation of add‐on therapies (LABA, leukotriene receptor antagonists, theophylline or anticholinergic agents) but did not require increased doses of ICS or oral CSs compared with normal‐weight counterparts.^58^ Interestingly, although obesity is linked to poorer responsiveness to ICS in older children and adults, a multi‐centre study involving 2–5 year‐old children has shown that preschool children with obesity‐related asthma do not exhibit reduced response to ICS therapy, suggesting that ICS should remain the first‐line treatment option for those population.^88^ Further research in CRS has shown that obesity is an independent risk factor for CSs insensitivity in patients with CRSwNP, particularly in those with eosinophilic CRSwNP.^91^ Impaired anti‐inflammatory response has also been observed in obese patients with AR during treatment with nasal CSs.^89^ The mechanisms by which obesity results in CSs insensitivity are briefly summarised as follows^4^, ^60^, ^90^, ^119^, ^120^, ^121^, ^122^: (1) obesity shifts the classical Th2‐type inflammatory profile to an exaggerated Th17‐type and neutrophilic inflammation; (2) proinflammatory environment may modify CSs function and decrease molecular responsiveness to CSs (reduced induction of mitogen‐activated protein kinase phosphatase‐1 expression); (3) abnormalities of CSs pharmacokinetics in obesity‐related allergic individuals; (4) other factors including airway mechanics, airway dysanapsis, lipid metabolites, genetics and so on may also contribute to CSs insensitivity. Interestingly, weight loss has been demonstrated to recover the CS sensitivity in obese asthmatics.^123^ Perhaps, patients with obesity‐related allergic diseases who are resistant to CSs should be encouraged to try weight loss before CSs administration.

However, whether decreased CS insensitivity is a cause or a result of obesity remains controversial. Since previous studies showed that patients with severe allergic airway diseases may require high doses and long‐term use of oral/local CSs aiming to control recalcitrant symptoms, which may, in turn, contributes to or exacerbates obesity.^124^, ^125^, ^126^ In this situation, reduced CS sensitivity may serve as a cause for obesity. Regardless of whether decreased CS sensitivity or obesity is the cause, achieving optimal disease control becomes less likely due to persistent inflammation and altered pulmonary mechanics.^4^, ^58^ Therefore, weight loss should be prioritised in the management of obesity‐related allergic airway diseases.

### 5.2 Biologics

Biological therapy represents a novel approach to treat severe and refractory patients with decreased response to conventional treatment. Biologics used in allergic airway diseases target T2 inflammation including anti‐IgE, anti‐IL‐4Rα, anti‐IL‐5/IL‐5Rα, anti‐thymic stromal lymphopoietin (TSLP) and so on (Figure 1).^127^ The impact of obesity on effectiveness of biologics in allergic patients is receiving growing attention.

#### 5.2.1 Omalizumab

Omalizumab, a humanised anti‐IgE monoclonal antibody, is currently recommended as add‐on therapy for patients with refractory airway inflammation. Obesity was identified as an independent factor reducing omalizumab response in severe asthmatics.^94^ In addition, a post‐marketing study including severe asthma population with significant comorbidities has shown that asthmatic patients with obesity are less likely to be categorised as responders than non‐obese patients, indicating reduced efficacy of omalizumab in obese asthmatics.^93^ In contrast, both Oliveira et al. and Geng et al. demonstrated that severe asthmatics with obesity treated with omalizumab displayed significant improvement in lung function and asthma control.^95^, ^96^, ^128^ The contradictory results may be attributed to the selection of patients with different immune endotypes. Interestingly, non‐atopic asthmatics with obesity who have been administrated omalizumab, display reduced emergency room visits, hospitalisations and CSs use.^129^ However, the investigators did not specifically examine the impact of obesity or increased BMI on the efficacy of omalizumab in patients with CRSwNP or AR. Thus, there is significant interest in assessing the specificity of omalizumab in the treatment of obese patients with CRSwNP or AR.

#### 5.2.2 Benralizumab and mepolizumab

Benralizumab is an anti‐eosinophilic, anti‐ IL‐5Rα humanised afucosylated monoclonal antibody that has been shown to significantly reduce asthma exacerbations and improve nasal polyp score for individuals with uncontrolled, severe asthma or CRSwNP.^97^, ^100^ BMI has been considered to a risk factor to predict poor response to benralizumab.^98^ And impaired efficacy of benralizumab was observed in severe, uncontrolled asthmatics with BMI > 35 as indicated by lower improvement in exacerbation rate.^97^ However, data from the SIROCCO and CALMA clinical trials demonstrated that benralizumab administration in obese asthmatics could exert beneficial functions based on the improvement in pre‐bronchodilator FEV_1_ and decreased number of asthma exacerbations.^99^, ^128^ Thus, it is unclear whether obese asthmatics could benefit from benralizumab. Of interest, phase 3 OSTRO trial (NCT0341229) studying the efficacy of benralizumab on CRSwNP showed that smaller nasal polyp score and nasal blockage score treatment effects were observed in patients with higher BMI compared with those with lower BMI,^100^ suggesting the influence of BMI on biologic response in CRSwNP is an intriguing area for further investigation. Although the treatment efficacy of benralizumab in obese allergic patients is conflicting, these data certainly pave a way to pursue further clinical trials evaluating the effectiveness of benralizumab in obese individuals with allergic respiratory diseases. The reduced efficacy of mepolizumab (anti‐IL‐5) in severe asthmatics with obesity was reported,^101^, ^102^ and the odds of achieving clinical remission decreased by 59% for those with obesity.^102^ While proof has shown that mepolizumab administration effectively reduces blood eosinophil counts and asthma exacerbations in obese asthma patients, the improvement in lung function, such as pre‐bronchodilator FEV_1_ or FVC% predicted, is lower compared with non‐obese patients.^103^, ^104^, ^105^ Although a phase III trial (SYNAPSE) has been performed in severe CRSwNP, the investigators did not evaluate the impact of BMI on the efficacy of mepolizumab in patients with CRSwNP.^130^ Obesity is associated with reduced efficacy, implying the potential importance of the treatable traits approach in addition to biologic therapy to achieve remission.

#### 5.2.3 Dupilumab

Dupilumab, a fully human IgG4 monoclonal antibody, blocks IL‐4/IL‐13 signal transduction that has been demonstrated to significantly improved asthma control and nasal polyp scores.^106^, ^108^ Previous data from the phase 3 QUEST trial (NCT02414854) demonstrated that dupilumab efficiently improved FEV_1_ and decreased the rate of severe asthma exacerbations regardless of BMI (dupilimab efficacy in patients with uncontrolled, moderate‐to‐severe asthma by BMI.^106^ Later, a long‐term (24 months) real‐life study displayed that obesity is a negative predictive factor for the efficacy of dupilumab in severe, eosinophilic asthmatics.^107^ However, the efficacy of dupilumab in obese CRSwNP seems not to be affected by BMI. As the proof (NCT02912468/NCT02898454) has shown that dupilumab administration (300 mg q2 W) efficiently improves objective, subjective and health‐related quality of life (QOL) in CRSwNP patients regardless of BMI.^108^

There are other miscellaneous biologics including tezepelumab (anti‐TSLP), secukinumab (anti‐IL‐17A), brodalumab (anti‐IL‐17Rα), etanercept (anti‐tumour necrosis factor alpha) and so on,^131^, ^132^, ^133^, ^134^ which are approved or undergoing clinical trials in allergic diseases. It is important to note that few of these studies stratified patients by BMI. Therefore, future studies focusing on the effectiveness of these biologics in obese patients with allergic airway diseases are warranted. More importantly, the poor response to biologics may be due to altered endotype, and it is imperative to identify therapeutic options for all existing endotypes of obese individuals. Since weight loss has been shown to improve hyporesponsiveness to CSs in obese‐related asthma,^123^ it is significant to evaluate whether weight loss can rescue the compromised efficacy of biologics in obesity‐related allergic diseases.

### 5.3 Allergen immunotherapy

Allergen immunotherapy (AIT) is a therapeutic vaccination approach to IgE‐mediated type I hypersensitivity diseases by inducing immunologic tolerance to allergens, which includes subcutaneous immunotherapy (SCIT), sublingual immunotherapy (SLIT) and other alternative routes.^135^ Recent studies have highlighted the impact of BMI on the efficacy and safety of AIT in AR patients. A study analysing the influencing factors related to the SLIT efficacy on Japanese cedar pollen challenged AR revealed that the effectiveness is diminished in AR patients with BMI ≥ 25 compared with those with BMI < 25.^109^ The result has been further confirmed in obese asthmatic patients undergoing SCIT, which demonstrated that lower improvement in FEV_1_/FVC is observed in obese asthmatics compared with those with normal weight.^111^ Increased BMI not only reduces the effectiveness of AIT, but also increases its adverse reactions. A study focusing on preschool children undergoing house dust mite SCIT reported that increased BMI is one of the risk factors for developing local adverse reactions.^112^ However, studies from Chinese populations receiving AIT have yielded inconsistent results, which found that BMI is not associated with efficacy of AIT.^110^, ^113^ These results suggest that obese children with allergic airway diseases could benefit from AIT regardless of BMI. However, the potential vulnerability of obese adults undergoing AIT may be present, and strategies such as dose adjustments and combinations of AIT and biologics may be implemented to achieve better outcomes. Similar studies with large cohorts and stratified BMI are required to comprehensively conclude about the impacts of obesity on the efficacy and safety of AIT, and to determine whether a combination of AIT and biologics should be implemented to achieve better outcomes.

### 5.4 Surgery

Endoscopic sinus surgery (ESS) is recommended to remove polypoid change and open the obstructed sinus to raise the efficacy of topical therapeutics delivery for severe CRS patients.^136^ Based on the evidence that obesity contributes to low‐grade systemic inflammation, researchers hypothesise that obese patients may display less postoperative improvement following ESS. A multi‐centre study demonstrated that obesity could reduce the relative improvement in QOL in CRS patients undergoing ESS.^114^ Consistently, a prospective study from Cleveland Clinic Foundation rhinology clinic has revealed higher postoperative sinonasal outcome test‐22 scores in obese CRSwNP patients, suggesting a reduced response to ESS in terms of symptom improvement.^34^ The same study also demonstrated a reduced postoperative response in extranasal sinus symptoms among CRS patients with obesity, which is possibly linked to the underlying cytokine profile.^34^ However, an analysis of the American College of Surgeons NSQIP database demonstrated that obesity does not increase the risk of 30‐day ESS postoperative complications.^115^ Obesity also affects recurrence rate post‐ESS. Three studies from Chinese demonstrated that increased recurrence rate is observed in overweight/obese CRS patients following ESS, particularly in non‐eosinophilic CRSwNP patients.^4^, ^76^, ^91^ However, due to the heterogeneity of CRS, the impact of increased BMI on recurrence in Western CRS patients warrants further exploration. Bronchial thermoplasty (BT) represents a non‐pharmacological treatment option for severe asthma.^137^ Some studies have shown its clinical effectiveness for obese asthmatic patients.^116^, ^117^ Unlike the potentially reduced efficacy of ESS in obese CRS patients, a cohort study stratified by BMI found that the asthma quality of life questionnaire scores were significantly improved in asthmatic patients with higher BMI compared with those with normal weight, indicating that severe asthmatic patients with high BMI may benefit from BT.^118^

## 6 THE POSSIBLE TREATMENT LANDSCAPE OF TOMORROW

Since obese patients with allergy usually respond poorly to traditional treatments, it is crucial to study novel therapeutic strategies for these patients (Table 2 and Figure 3).

**TABLE 2:** Promising therapies to obese patients with allergic airway inflammation.

Intervention	Diseases	Mechanisms	Types of research	Mentioned studies
**Weight loss shared mechanisms**				
Diet	Asthma	Losing weight Anti‐inflammation Regulating the gut microbiota Improving metabolic conditions Decreasing AHR	Feasibility RCT	Sharma et al.^138^
			Crossover design pilot trial	NCT05222451
			Stratified RCT	NCT05980663
Exercise	Asthma		RCT	Freitas et al.^139^
			Prospective, three‐arm RCT	NCT06326632
Diet and exercise	Asthma		RCT	Türk et al.^140^
Bariatric surgery	Asthma		Prospective study	Dixon et al.^141^
			Longitudinal cohort study	van Huisstede et al.^142^
			Prospective controlled parallel group study	Boulet et al.^143^
			Pilot study	Maniscalco et al.^144^
Diet, exercise and intragastric balloon device	Asthma		Multicentre RCT	NCT05364957
**Microbiome‐targeted therapy**				
Probiotics	Asthma	Impacting on the resident microbiota and shaping the immunological landscape	Murine model	Michalovich et al.^145^
			Pilot study	NCT05949255
Prebiotics	Asthma		Murine model	Tashiro et al.^146^
**GLP‐1R agonists**				
GLP‐1R agonists	Asthma	Inhibiting allergic airway inflammation, decreasing airway eosinophilia, mucus production and hyperresponsiveness	Murine model	Toki et al.^147^
			Retrospective cohort study	Foer et al.^148^
			Phase II trial	NCT05254314
**Others**				
l‐citrulline	Asthma	Preventing asymmetric dimethyl arginine‐mediated NO synthase uncoupling, restoring NO and reducing oxidative stress	Phase II proof‐of‐concept trial	Holguin et al.^149^
			Phase II trial	NCT03885245
			Pilot trial	NCT02943161
Vitamin D	Asthma	Regulating immune system and anti‐inflammation	Prospective, open label RCT	O'Sullivan et al.^150^
			Double‐blind, parallel (2 arms) RCT	NCT05431920
1‐O‐alkyl‐glycerols	Asthma	Being involved in the synthesis of plasmalogens, reducing inflammation and oxidative stress	Parallel, controlled clinical trial	Denisenko et al.^151^
MitoQuinone mesylate	Asthma	A mitochondrial antioxidant reducing allergen‐induced inflammation, AHR, mucus hypersecretion, airway fibrosis and protein oxidation	Murine model	Chandrasekaran et al.^152^
			Phase I trial	NCT04026711
Platycoside E	Asthma	One of the Fermented Platycodon grandiflorum extracts, relieved AHR and airway inflammation	Murine model	Xu et al.^153^
Oral tolerance	Allergic airway diseases	Maintaining and expanding the M1 macrophages, reducing the Th2‐mediated allergic responses	Murine model	Geissler et al.^2^

Abbreviations: AHR, airway hyperresponsiveness; GLP‐1R, glucagon‐like peptide‐1 receptor; NO, Nitric Oxide; RCT, randomised controlled trial.

### 6.1 Weight loss: diet restriction, exercise, intragastric balloons and bariatric surgery

Growing clinical evidence has shown that weight loss by non‐surgical and surgical approaches contributes to alleviating the severity of obesity‐related inflammation in patients.^154^ A randomised, controlled trial in European adults demonstrated that targeting refractory asthma associated with obesity using total diet replacement weight management results in clinical improvements in asthma‐related symptoms and QOL with significant reduction of body weight.^138^ Additionally, the effect of weight reduction achieved by exercise training on asthma control has been studied. Adding exercise to a short‐term weight loss program achieved greater weight loss, with subsequent significant improvements in lung function and anti‐inflammatory biomarkers, as well as reductions in airway and systemic inflammation in obese patients with asthma.^139^ Moreover, another European clinical trial involving 34 obese asthmatics showed that intervention using a high—intensity pulmonary rehabilitation program significantly decreased body weight, with improvement of asthma control and aerobic capacity compared with usual care.^140^ Therefore, weight loss by diet and/or exercise intervention has well‐defined benefits in improving respiratory pathophysiology and should be recommended by physicians. However, as a stand‐alone therapy, life interventions have limited efficacy and durability. Intragastric balloons (IGBs), non‐surgical and minimally invasive devices that alter appetite, are the most widely available endoscopic bariatric therapy for obesity (class I and II).^155^ Patients treated with IGBs are 1.94–4.31 times more likely to achieve 5% and 10% total body weight loss than those receiving lifestyle therapy alone.^155^ There is limited evidence from RCT data for the efficacy of IGB for the treatment of obesity‐related allergic airway diseases; however, clinical trials (NCT05364957) evaluating the effectiveness of IGBs in obese patients with asthma are undergoing in France. We hope that results from ongoing clinical trials will provide positive outcomes, offering new treatment options for obesity comorbidities. Additionally, obese asthmatics who underwent bariatric surgery displayed significant improvement of asthma control, AHR, medication use and hospitalisation rate, along with weight loss.^141^ ^142^, ^156^ ^,^ However, the inconsistency in the effects on AHR may be attributed to variations in surgical procedures and distinct baseline characteristics among patients such as IgE levels.^141^, ^142^, ^143^ More importantly, the efficacy might be persistent, as indicated by improvement of asthma‐related symptoms and QOL at a 5‐year follow‐up after surgery in asthmatic patients with severe obesity.^144^

Of note, lifestyle modifications, such as dietary restriction and exercise training, remain the most accessible approach for obese patients with allergy and should be prioritised. Although the evidence is limited, concomitant or sequential use of high‐intensity lifestyle interventions, IGBs and bariatric surgery has been related with more significant and sustained weight loss. The potential concomitant or sequential therapies may be used as adjunct interventions for patients with obesity‐related allergic diseases in clinical practice.

### 6.2 Microbiome‐targeted therapy: probiotics and prebiotics

Gut microbiota has been shown to systemically shape the immunological landscape.^157^ Gut microbiome‐targeted therapies including faecal microbial transplantation, prebiotics and probiotics are currently among the treatments being considered for obesity‐related allergic airway diseases.^10^ Decreased abundance of faecal Akkermansia (A.) muciniphila has been observed in severe asthmatics with obesity and administration of A. muciniphila to murine models significantly reduces AHR and airway inflammation.^145^ Despite the fact that the efficacy of A. muciniphila in humans remains unevaluated, bariatric surgery induces a significant shift of the gut microbiome with increased abundance of A. muciniphila.^158^ These results suggest that administration of A. muciniphila may induce a beneficial effect for obese asthmatics. Prebiotics such as dietary fibre have the capacity to alter the gut microbiome and attenuate allergic airway inflammation. Experimental data demonstrated that dietary supplementation with fermentable fibre pectin could alleviate ozone‐induced AHR and neutrophilic airway inflammation with a reduction of IL‐17A.^146^ These data imply that the use of prebiotics is a promising candidate for gut microbiome‐targeted therapy for allergic airway inflammation with obesity.^154^

### 6.3 Glucagon‐like peptide‐1 receptor agonists

Glucagon‐like peptide‐1 receptor (GLP‐1R) agonists are approved to treat type 2 diabetes mellitus and obesity. Increasing experimental evidence has shed light on the key role of GLP‐1R agonists in the treatment of allergic airway inflammation in preclinical models.^148^ Toki et al.^147^ demonstrated that GLP‐1R agonist administration could blunt neutrophilic airway inflammation by inhibiting ILC2 response in obese mice with allergic inflammation. A recent retrospective study showed that adult patients with asthma treated with GLP‐1R agonists for their type 2 diabetes had lower rates of asthma exacerbation than those treated with other diabetic drugs.^148^ In addition to GLP‐1R single agonists, preclinical and clinical trials have demonstrated the efficacy of GLP‐1R/glucagon receptor (GCGR) co‐agonists, GLP‐1R/glucose‐dependent insulinotropic polypeptide receptor (GIPR) co‐agonists and GLP‐1R/GIPR/GCGR triagonists for obesity treatment,^159^ which holds great promise for the future of anti‐obesity‐associated allergic airway diseases.

### 6.4 Other possible treatments

Beyond the novel strategies mentioned above, other treatments including dietary supplements and antioxidants may also hold promise for managing obesity‐related allergic diseases: (1) A proof‐of‐concept, open‐label pilot study displayed that short‐term l‐citrulline treatment (15 g/d) was found to increase FeNO levels and improve asthma control in obese asthmatics.^149^ (2) A systematic review suggested potential benefits of vitamin D supplementation for obesity‐related asthmatic children,^160^ and relevant clinical trials are ongoing.^150^ (3) Intake of 1‐*O*‐alkyl‐glycerols (AGs) led to improved lung function and increased concentration of plasmalogen and n‐3 polyunsaturated fatty acid in obese asthmatics suggesting AGs may be a beneficial dietary supplement for alleviating inflammation and improving lung function in individuals with obesity‐related asthma.^151^ (4) Mitoquinone mesylate targeting mtROS was found to attenuate mtROS–NLRP3 inflammasome mediated airway inflammation and hyperresponsiveness in an obese murine model of allergic airway disease and human normal bronchial epithelial cell lines BEAS‐2 cells,^51^, ^152^ suggesting that anti‐mtROS and small molecule NLRP3 inhibitors may be candidates for treating obesity‐related airway inflammation. (5) Alleviated allergic airway inflammation was also found in an experimental model of obese asthma in mice treated with platycoside E which is extracted from platycodon,^153^ suggesting efficacy of traditional herbal medicine for treating obesity‐related airway inflammation. (6) Oral tolerance induction could effectively attenuate the enhanced allergic airway inflammation in obese mice with allergy,^2^ implying further research is needed to confirm the potential therapeutic effects on obese patients with allergic airway diseases.

## 7 ARE ALLERGIC AIRWAY DISEASES A RISK FACTOR FOR OBESITY?

Recently, increasing clinical evidence suggests that allergic airway diseases may also conversely contribute to the development of obesity. Several multicohort studies from the United States and Europe have shown that both asthmatic children and adults have a higher risk for obesity incidence than non‐asthmatic counterparts.^161^, ^162^, ^163^, ^164^ Additionally, a causal association between CRSwNP and subsequent obesity has also been identified (hazard ratio (HR): 1.74, 95% CI: 1.08–2.80),^126^ while this link appears weaker in childhood AR (adjusted HR: 1.29, 95% CI: 0.98–1.68),^162^ These may be attributed to long‐term use of higher doses of oral CSs and lack of physical activity among those patients, though the exact mechanisms remain to be fully addressed.^126^, ^163^, ^164^ Of note, the use of asthma rescue medications could reduce the risk of developing obesity by 43 percent, possibly because of the direct effect of the β‐agonists on the adipocytes.^161^ Although there is a debate about whether obesity or allergy is the ‘chicken’ or the ‘egg’, undoubtedly, the bidirectional causal relationship will contribute to a vicious cycle of worsening obesity and allergic diseases. Therefore, further research is needed to fully understand the bidirectional association between allergic diseases and obesity, including whether early intervention is required to prevent the vicious cycle.

## 8 CONCLUSIONS AND FUTURE DIRECTIONS

**FIGURE 4:**
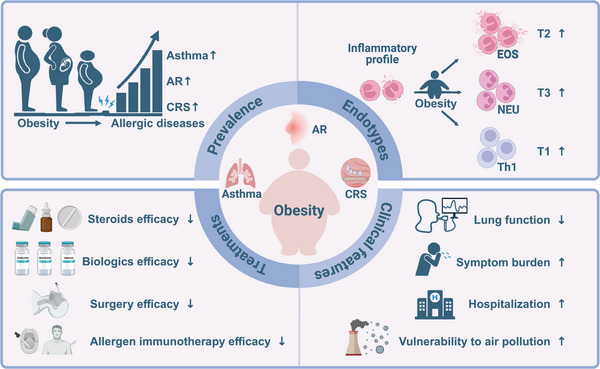
The impact of obesity on allergic airway diseases. Obesity affects various aspects of allergic airway diseases, including prevalence, endotypes, clinical features and therapeutic efficacy. The prevalence of allergic airway diseases such as asthma, AR and CRSwNP can be increased by obesity. Obesity alters the immune endotype and exacerbates clinical symptoms of respiratory allergic diseases. Obesity‐related allergic airway diseases exhibit therapeutic resistance to standard treatment. Therefore, obesity‐related allergic airway diseases represent a unique class of endotypes and phenotypes, which need much more in‐depth research and clinical trials as well as therapeutic approaches. AR, allergic rhinitis; CRSwNP, chronic rhinosinusitis with nasal polyps; EOS, eosinophils; NEU, neutrophils; T1, type 1; T2, type 2; T3, type 3; Th1 cells, CD4+T helper 1 cells. Figure was created with BioRender.

The present review showed the impact of obesity on allergic airway diseases, focusing particularly on prevalence, clinical symptom burden, immune endotype and therapeutic resistance based on clinical and preclinical studies (Figure 4). As current treatments are less effective in obese patients, possible therapeutic interventions such as weight loss, gut microbiome‐targeted treatment, GLP‐1R agonists and other agents were specifically addressed. Lifestyle interventions are a cornerstone of obesity management for patients with obesity‐associated allergic airway diseases, and weight loss should be prioritised. However, as a stand‐alone therapy, lifestyle interventions have limited effectiveness and durability. Therefore, the future of obesity management may encompass the full spectrum of interventions from lifestyle changes, medications, IGBs and bariatric surgery. Of note, several key questions remain to be answered: (1) The bidirectional cause‐effect relationship between allergy and obesity will contribute to a vicious cycle, whether weight loss and anti‐allergy therapy can break this loop and significantly improve disease control? (2) For patients with obesity‐related allergic airway diseases who respond poorly to T2 biologics, CSs or AIT, whether weight loss can improve or restore treatment efficacy? If so, weight loss should be recommended before administering these medicines. (3) Further research will also focus on personalised prognostic and predictive biomarkers that will enable physicians to personalise treatment choice to maximise efficacy. Much more in‐depth basic and clinical work is needed to fully understand the interaction between obesity and allergic airway diseases and to develop novel tailored preventive and therapeutic strategies.

## AUTHOR CONTRIBUTIONS

Yana Zhang designed the study and approved final submission. Wenlong Li and Noah Marx drafted the manuscript. Yana Zhang, Deyu Fang and Qintai Yang critically revised the manuscript.

## CONFLICT OF INTEREST STATEMENT

The authors declare no conflicts of interest.

## ETHICS STATEMENT

Ethical approval is not applicable to this study.

## Data Availability

Data sharing is not applicable to this article as no new data were created or analysed in this study.
